# P16INK4A—More Than a Senescence Marker

**DOI:** 10.3390/life12091332

**Published:** 2022-08-28

**Authors:** Hasan Safwan-Zaiter, Nicole Wagner, Kay-Dietrich Wagner

**Affiliations:** CNRS, INSERM, iBV, Université Côte d’Azur, 06107 Nice, France

**Keywords:** aging, cancer, development, p16, pathologies, senescence

## Abstract

Aging is a biological feature that is characterized by gradual degeneration of function in cells, tissues, organs, or an intact organism due to the accumulation of environmental factors and stresses with time. Several factors have been attributed to aging such as oxidative stress and augmented production or exposure to reactive oxygen species, inflammatory cytokines production, telomere shortening, DNA damage, and, importantly, the deposit of senescent cells. These are irreversibly mitotically inactive, yet metabolically active cells. The reason underlying their senescence lies within the extrinsic and the intrinsic arms. The extrinsic arm is mainly characterized by the expression and the secretory profile known as the senescence-associated secretory phenotype (SASP). The intrinsic arm results from the impact of several genes meant to regulate the cell cycle, such as tumor suppressor genes. P16^INK4A^ is a tumor suppressor and cell cycle regulator that has been linked to aging and senescence. Extensive research has revealed that p16 expression is significantly increased in senescent cells, as well as during natural aging or age-related pathologies. Based on this fact, p16 is considered as a specific biomarker for detecting senescent cells and aging. Other studies have found that p16 is not only a senescence marker, but also a protein with many functions outside of senescence and aging. In this paper, we discuss and shed light on several studies that show the different functions of p16 and provide insights in its role in several biological processes besides senescence and aging.

## 1. Introduction

P16 is a tumor suppressor gene, which has been termed with several names such as the multiple tumor suppressor-1 (MTS-1), the inhibitor of cyclin-dependent kinase 4a (INK4A), or the cyclin-dependent kinase inhibitor 2a (CDKN2A). The human p16 gene is located on the short arm of chromosome (9p21.3). The p16 transcript is composed of three exons which encode 156 amino acids. The use of an alternative reading frame generates the human p14Arf protein (p19Arf in mice) [1,2,3].

Since its discovery, p16 has grown a significant importance in the aging and cellular senescence domain. It has formed the bedrock for demonstrating aging and cellular senescence processes and their correlation with other tumor suppressors and cell cycle regulators such as p21 and p19ARF [4,5,6]. Several studies have shown that p16 has a dramatically increased expression in a variety of tissues of old rodents, including the lungs, lymph nodes, adrenal gland, and uterus. Furthermore, removing p16-positive senescent cells delayed the occurrence and progression of age-related pathologies in mice in vivo, as well as prolonging the lifespan of premature and natural aging mice [7,8]. All these combined made p16 a hallmark of aging and age-related diseases.

Aging is the common risk factor for several diseases [9] that contribute to a high rate of global morbidity and mortality [10,11,12,13,14,15,16,17]. Different factors contribute to aging, including accumulated oxidative stress and reactive oxygen species production, increased proinflammatory cytokine production, DNA damage, telomere shortening, and oncogene activation [18,19,20,21,22]. These molecular and biochemical mechanisms promote aging through accumulation of senescent cells in tissues. Whether these different stresses promote cellular senescence through a common pathway or distinct mechanisms remains an open question.

Cellular senescence is an irreversible proliferative arrest caused by two major intrinsic and extrinsic stimuli in response to potentially oncogenic stress [23]. It was first demonstrated in cell culture, when cells ceased growth after several passages, and it was termed the Hayflick’s limit [24]. Microscopically, senescent cells are typically larger than normal cells of the same type, have a flattened shape, and occasionally have multiple nuclei. However, analyzing the expression profile of these cells revealed a number of senescence-related genes [25,26,27,28]. The senescence-associated β-galactosidase (SA β-gal) is a typical biomarker for the detection of senescence. P16 and p21 are also frequently associated with senescence. Moreover, the senescence-associated secretory phenotypes (SASPs), which are a mix of proinflammatory cytokines, proteases, and growth factors, are upregulated and secreted by senescent cells and are part of the extrinsic arm of cellular senescence [29]. As no single reliable marker for the detection of senescence in vivo exists, the recent consensus from the International Cell Senescence Association (ICSA) requests at least a combination of more than two markers to identify a cell as senescent [30].

P16 along with other tumor suppressors characterize the intrinsic arm of cellular senescence by forming two pathways. The p16–pRB where p16 acts as an upstream regulator of pRB and the p53–p21 where p21 is a downstream effector of p53 [3,31,32,33]. The major regulatory key of these pathways is the inhibition of the cyclin-dependent kinases CDK4/6 and CDK2 which then control cell cycle and implicate them in physiological processes dependent on cellular proliferation such as cancer, tissue regeneration, aging, and cellular senescence and development [32,34,35]. As a result, p16 upregulation or baseline expression is not solely associated with aging and senescence, but it may simply reflect cell cycle inhibition as p16 upregulation is not always associated with increased SASP expression [36]. In addition, p16 suppression restores proliferation and provokes senescence bypass associated with decreased SASP expression [37,38] and promotes tumorigenesis [39,40,41,42].

Recent studies have demonstrated an increased p16 expression after injury; p16 positive senescent cells are critical for tissue repair and wound healing. These cells aided in wound healing and tissue repair, but their removal resulted in delayed cutaneous wound closure [43]. Additionally, p16 coexpression with laminin 5 was found indispensable for growth arrest of migratory keratinocyte during wound re-epithelialization [44]. Similarly, fibro-adipogenic progenitors (FAPs) exhibited transient p16 upregulation in response to acute muscle injury (AMI) [45]. On the other hand, p16 regulates stem cell self-renewal processes in a variety of tissues, and its disruption may result in aging or tumor development [46]. Moreover, induced liver damage in p16- and p53-deficient mice results in increased fibrosis [47]. A recent study demonstrated a role of p16 as a potential therapeutic target for intervertebral disc damage. The level of p16 expression in intervertebral discs correlated with the severity of tissue damage in humans and mice. P16-deficient mice showed attenuated intervertebral disc degeneration in their model [48].

In embryonic development, a potential role of p21 and of p19ARF has been unraveled [34,49,50,51,52], but little is known about p16. Its role in development requires further elucidation [53,54,55]. Therefore, most of what we know about p16 is restricted to senescence, aging, and diseases [29]. In this review, we discuss the effects of p16 in several tissues, as well as its potential role in embryonic development and liver physiology, which was recently demonstrated.

## 2. The P16 Gene

**Figure 1:**
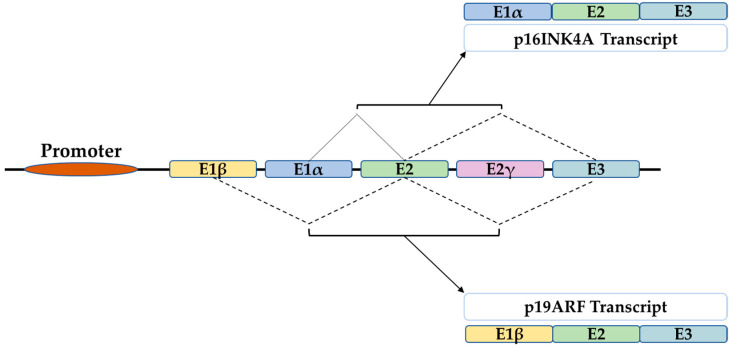
The structure of the INK4A locus gives rise to several transcripts through alternative splicing. P16INK4A and p19ARF are two transcripts of 3 exons which differ only in the first exon that is E1α for p16 and E1β for p19ARF.

The *CDKN2A gene* belongs to the *INK4 genes* family. *CDKN2A* encodes for p16Ink4A and p14ARF (p19Arf in mice), while *CDKN2B* encodes for p15Ink4B, *CDKN2C* for p18Ink4D, and *CDKN2D* for p19Ink4D. They share biological properties in cell cycle regulation and tumor suppression [56,57]. The p16*^INK4A^* structure consists of five exons E1β, E1α, E2, E2γ, and E3. Alternative splicing generates four different transcript variants including p16 (E1α, E2, and E3), p19Arf (E1β, E2, and E3), (the murine orthologue of the human p14ARF), in addition to p16γ and p12. Thus, the difference between p16 and p19ARF transcript variants lies within the alternative splicing of E1α versus E1β [58,59] (Figure 1).

## 3. P16 Function and Regulation of Expression

The cell cycle is a complex loop of events consisting of doubling the genetic material of the mother cell in the S phase, which is afterward accurately segregated into two identical daughter cells in the M phase. Once out of the quiescent G0 phase and prior to the S phase, the cell enters a critical preparatory gap phase termed G1. There, the cell fate hinges to decide at this restriction point whether to progress through the cycle. Generally, the driving forces of progression through the cell cycle are the regulatory cyclin-dependent kinases (CDKs). CDKs are activated by binding to their corresponding cyclins. Specifically, at the G1/S checkpoint, the progression through the cell cycle requires the cyclin D–CDK 4/6 assembly [60,61].

**Figure 2:**
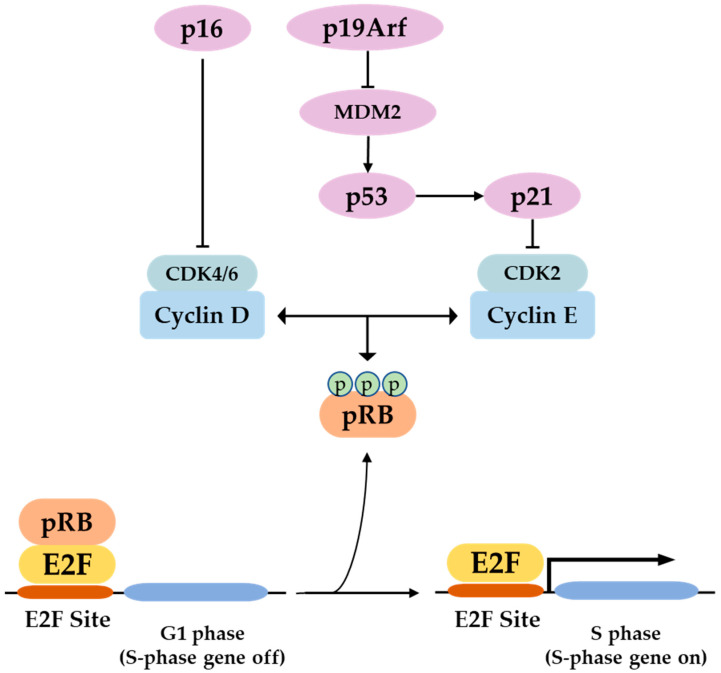
In the p16/pRB pathway, p16 inhibits Cdk4/6–cyclin D complex formation and induces subsequent pRB hypophosphorylation. Similarly, in the p53/p21 pathway, p19ARF traps MDM2 and prevents p53 degradation, which consequently activates p21. This works similarly as p16 but by inhibiting Cdk2–cyclin E complex, and, therefore, induces pRB phosphorylation. Dephosphorylated pRB binds E2F transcription factor at the E2F sites and blocks G1–S phase transition, blocking the cell cycle. However, in the absence of p16 and p21, hyperphosphorylated pRB detaches from E2F transcription factors, which consequently activates S-phase genes and induces progression of the cell cycle.

P16 is a specific inhibitor of the cyclin-dependent kinases CDK4 and CDK6 and it mainly prevents cell transition from G1 to S phase and causes subsequent proliferation arrest by rendering retinoblastoma protein (pRB) in a hypophosphorylated state. In G0, pRB is unphosphorylated. At the beginning of G1, cyclin D-Cdk4/6 monophosphorylates pRB, which is bound to E2F transcription factors. When the cell passes the restriction point, cyclin E-Cdk2 hyperphosphorylates pRB, which dissociates from the E2F factors, which in turn translocate to the nucleus and activate transcription of S phase genes [3,33,58,62]. However, in an alternative model proposed by Ahlander and Bosco, hypophosphorylated pRB binds E2F transcription factors on the chromatin, and recruits histone deacetylase (HDAC) and other chromatin-remodeling enzymes, which act all together to inhibit S-phase gene transcription and cell cycle progression. On the contrary, hyperphosphorylated pRB dissociates from E2F which in turn activates transcription and cell cycle progression [63]. P16 binding to CDK 4/6 can directly inhibit their activities in addition to non-p16 CDK inhibitors such as p27 for further hypophosphorylation of pRB (Figure 2) [64]. This p16–pRB–E2F axis can also be enhanced through direct interaction of p16 with the gene-associated retinoid-IFN-induced mortality-19 (GRIM-19) [65].

Hereby, p16 expression seems to be credited to a feedback loop with pRB. In other words, pRB phosphorylation provokes E2F activation and induces p16 expression. Thus, p16 inhibits CDK 4/6 and increases hypophosphorylated pRB which tends to downregulate p16 [66]. P16 expression can be affected by epigenetic modification through promoter hypermethylation [67]. PRC1 and PRC2 complexes are involved in this response and provoke heterochromatin formation and p16 suppression [68,69]. The hypermethylation is mediated by the PRC2 core protein Ezh2. Ezh2 adds a trimethyl to H3K27 in an association with the polycomb protein Bmi-1, a member of the PRC1 complex. Bmi-1 dissociation from the suppression complex can restore p16 expression [70,71]. In addition, as a major cause of cellular senescence and aging, both exogenous- and endogenous-induced oxidative stress and reactive oxygen species (ROS) production resulted in upregulation of p16 expression in pathways that involve the extracellular signal-regulated kinases ERK1/2 and the stress-activated protein kinases p38 [72,73].

Furthermore, several studies have shown other mechanisms of p16 inhibition of the cell cycle independently from the pRB–E2F pathway [74,75]. One in which p16 interacts with the CDK7 subunit of the general transcription factor TFIIH to inhibit the phosphorylation of the carboxyl–terminal domain (CTD) of the large subunit of the RNA polymerase II, contributing to cell cycle arrest [74,76]. Furthermore, it also inhibits the activities of the c-jun N-terminal kinase (JNK1 and JNK3) which in turn blocks AP-1 activity and inhibits cell transformation [75].

Moreover, the case of oncogene-induced senescence through elevated p16 expression was observed in primary cells with activated RAS or its downstream stream effectors Raf and MEK [77,78]. More precisely, the Ets family members, Ets1 and Ets2, which are transcription factors activated by the RAS–Raf–MEK cascade [79], bind and activate the p16 promoter with 5- to 10-fold potentiation [80]. Independently, RAS can also increase p16 expression, and induce cellular senescence by inducing the ectopic expression of the transcription factor HBP-1 [81] or activation of the H3K27 histone demethylase JMJD3 and downregulation of Ezh2 [82].

## 4. Role of p16 in Different Tissues and Organs: Cancer, Physiology, and Pathophysiology

In several selected tissues and organs, including skin, bones, lungs, brain, heart, kidney, and liver, we intend to address the well-known function of p16 in senescence and aging, and discuss several functions of p16, which might be more related to its classical role as a cell cycle regulator. Multiple studies investigating p16 functions used the p16-INK-ATTACK [7], p16-3MR [43], p16-Cre [83], and Super-INK4A/ARF mice. The p16-INK-ATTACK transgene consists of a 2.6 kb p16 promoter construct controlling expression of green fluorescent protein (GFP) and a FKBP–Casp8 “killing cassette”, which upon treatment with AP20187 induces apoptotic death of FKBP–Casp8 expressing cells [7]. GFP expression in these animals increased significantly between 12 and 18 months of age [8]. The p16-3MR transgene consists of a 50 kb p16 BAC clone containing the 3MR (trimodality reporter) fusion protein, with a synthetic Renilla luciferase (LUC), monomeric red fluorescent protein (mRFP), and truncated herpes simplex virus 1 thymidine kinase (HSV-TK). HSV-TK allows the killing of positive cells by ganciclovir. A significant increase in luciferase activity was detected by 18 months of age [43]. P16-Cre mice represent knock-in models in the endogenous p16 locus either as constitutively active Cre or Tamoxifen-inducible CreERT2 versions. These mice were crossed with mTmG reporter of diphtheria toxin deleter strains. Some GFP-positive cells were detectable by 2 months of age and the number increased significantly at 12 months [83]. The Super-Ink4a/Arf mouse strain carries an additional transgenic copy of the entire Ink4a/Arf locus [84]. Recently, p21-CreERT2 [85] and p21-INK-ATTACK [86] mice were established. Data from these animals suggest that p16 and p21 mark different populations of senescent cells and have different functions. Additionally, the SASPs of p16 and p21 cells are functionally different [87]. Most of the studies showed that elimination of the small number of senescent cells improves tissue health [7,8,86,88,89,90,91,92,93,94,95,96], but some studies also provided evidence for a physiological function of senescent cells and the SASP [43,83]. As beneficial effects were observed in tissues where the p16-INK-ATTACK transgene is not expressed [8,91], p16 knockdown inhibits SASP factor expression [37], and very recently it has been shown that blood or plasma transfer from old to young mice induces senescence and aging features [97], it is likely that the SASP plays the most important role for the observed alterations. Studies related to p16 function in different organs are detailed below.

### 4.1. In the Skin

The skin is the largest tissue in the human body. It serves as a physical barrier to both biological and nonbiological threats. Being exposed to the outside environment places the skin in direct contact with environmental hazards, making it extremely vulnerable. The skin is made up of two layers: the outer epidermis, which is divided into four sublayers with keratinocytes predominating in the spinous, granular, and cornfield sublayers, and pigment-producing melanocytes that confer photoprotection in the basal sublayer. The underlying dermis contains connective tissue with fibroblasts, collagen, and elastin as well as sebaceous and sweat glands and is connected to the epidermis by the dermal epidermal joint (DEJ) [98]. Skin aging is caused by both intrinsic (genetic, time, etc.) and extrinsic (pollution, UV exposure, sunlight, etc.) factors, and it has both biological and functional implications. Aged skin has thinner epidermis, dermis, and DEJ than younger skin, which is due to keratinocytes’ decreased proliferation and renewal ability [25,99,100,101].

As major biomarkers of senescence, both the SA-β-gal and p16 determination has shown elevated expression upon in vitro exposure of fibroblasts and keratinocytes to UV light [102,103,104]. Furthermore, telomere shortening, DNA damage, and UV exposure increased the activity of the p16/pRB and P19ARF/p53/P21 cascades, resulting in an accumulation of senescent cells and skin stem cell dysfunction and loss of regeneration capacity [101]. An in vivo study, on the other hand, claimed that UV light exposure has accelerated cellular senescence by increasing p21 expression [104].

In contrast to the previous results, the presence of p16 has been shown to play an important role in several biological processes that are beneficial to the skin. Starting with its tumor suppression function, p16 inactivation due to mutation or promoter methylation has been linked to a variety of cancers, including familial and sporadic melanoma [105,106,107,108]. These studies identified 55 out of 60 melanoma cell lines that were dependent on complete or partial p16 aberration, implicating this pathway in the development of melanomas. Furthermore, the level of p16 expression could be used as a melanoma predictive and prognostic biomarker. In other words, lower p16 levels were associated with higher Ki67 expression as a proliferation marker, and metastatic melanoma lesions were associated with even lower p16 levels and predicted poor patient survival [109]. Benign nevi had higher p16 levels than nonmetastatic melanoma, which had even higher p16 levels than metastatic melanoma [110]. Furthermore, in primary mouse fibroblasts (PMFs), human melanocytes, and a human melanoma cell line (A375), the loss of p16 correlated with increased mitochondrial mass, attenuated respiration, and altered morphology associated with augmented superoxide production and higher cellular motility. Forced p16 expression restored mitochondrial homeostasis, dynamics, and motility in a CDK4/pRB independent pathway [111]. Surprisingly, oxidative stress-induced p16 has attenuated ROS production in skin in vivo and in vitro. In addition, elevated intracellular ROS and DNA damage were obtained in p16-deficient cells. This was restored in skin fibroblasts transduced with p16 using lentivirus [72]. These findings suggest a pRB-independent tumor suppression function of p16. As another mechanism, p16 has been found to transactivate the promoter of the tumor suppressor miRNAs, miRNA-141 and miRNA-146b-5p, in melanocyte through physical interaction with the transcription factor Sp1 and CDK4, via the p16 fourth ankyrin repeat. Mutation in this ankyrin repeat attenuated Sp1 binding and miRNA-141 and miRNA-146b-5p transactivation without affecting the expression level of Sp1 [112]. In addition, this p16–Sp1–CDK4 interaction and consequent miRNA-141 and miRNA-146b-5p transactivation has also been implicated in cellular response to UV-radiation-induced damage and apoptosis.

P16 has been shown to be an important factor in wound healing. Endothelial cells and fibroblasts were identified as p16-positive cells at the site of injury in the p16-3MR mouse model a few days after injury. These transiently appearing senescent cells aimed to accelerate wound closure by inducing myofibroblast differentiation via platelet-derived growth factor AA secretion as part of the SASP [43]. Elimination of these cells delayed the wound healing process. The matricellular protein CCN1 has been identified as a key player in the induction of fibroblast senescence at the wound healing margins. By inducing DNA damage and p53 activity, CCN1 induces oxidative stress and provokes p16 upregulation, which leads to fibroblast senescence and antifibrotic gene activation [113]. Furthermore, coexpression and activation of the laminin 5/p16 response has been identified in migrating keratinocytes. The laminin 5/p16 response caused hypermotility and growth arrest in keratinocytes, leading to wound re-epithelialization [44]. This pathway has also been identified in critical stage neoplastic progression as a tumor suppressing pathway. This might suggest a protective effect of the induced p16 upregulation upon the exposure of skin to UV radiation [114].

**Figure 3:**
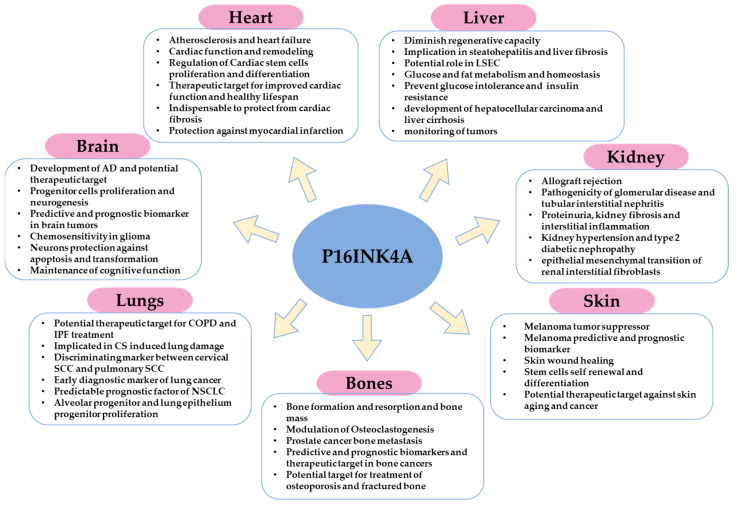
Schematic illustration that summarizes the major functions or implications of p16 in homeostasis, pathophysiology, and cancer of different organs.

Moreover, p16-orchestrated expression is required for stem cell self-renewal and differentiation. More precisely, p16 repression by epigenetic regulators is indispensable for stem cells proliferation. On the contrary, its promoter epigenetic regulation and orchestrated expression level have been found crucial for keratinocyte differentiation beside many other differentiation genes [115,116,117,118,119]. However, p16 seems not causal for terminal differentiation as it is expressed during early embryonic development [120], but still the balance between growth and differentiation requires a balanced expression of p16 and other cell cycle regulators [116,117,118,119]. For instance, Id-1, Id-2, and Id-3 are repressors of p16 and are upregulated in dividing keratinocytes, whereas they become downregulated in differentiated cells [121]. Activators of p16 transcription promoted keratinocyte differentiation via acting on epidermal differentiation complex genes [46]. Therefore, unravelling the precise mechanism underlying p16 regulation of expression might provide a targeted approach which confers maintenance of epidermis regenerative capacity and avoids premature skin aging or cancer development (Figure 3 and Table 1).

### 4.2. In the Bones

Two major types of cells are involved in maintaining skeletal homeostasis: osteoblasts, which are derived from osteoprogenitor cells and are in charge of bone growth, mineralization, and remodeling, and osteoclasts, which are descended from myeloid lineages and mediate bone resorption and breakdown [122]. Osteocytes are the most prevalent long-lived cell type in bone matrix and are in charge of maintenance of bone mass [123]. Skeletal aging is characterized by bone mass loss and is a significant risk factor for osteoporosis because it results from an increase in osteoclasts and a decrease in osteoblasts count [124,125,126,127]. Cellular senescence has been linked to bone aging and the development of aging-related osteo-pathologies [128]. More precisely, senescent osteocytes have been detected in aging bones with increased expression of p16.

In contrast to osteocytes, senescent osteoblasts are characterized by increased expression of p21 only [123,126,129,130,131]. Moreover, the selective elimination of p16-expressing cells using INK-ATTACK transgene resulted in increased bone mass in 20 months old mice [132]. Furthermore, using the p16-3MR transgene, which is based on the elimination of P16-expressing cells upon treatment with ganciclovir (GCV), has effectively abrogated age-related increases in osteoclastogenesis of the myeloid lineage but had no effect on bone formation. This might indicate that p16, rather than direct targeting of senescent osteocytes, contributes to osteoclastogenic potential without major impact on age-related bone loss [133].

However, other implications of p16 have been demonstrated in bone. P16 degradation by the ubiquitinated regulator UBE2S is an important step in the progression of prostate cancer bone metastasis [134]. Furthermore, patients with p16-positive oropharyngeal squamous cell carcinoma had a higher incidence of bone metastasis than p16-negative patients [135]. Lower expression of p16 in osteosarcoma patients was correlated with reduced response to primary chemotherapy [136], which, therefore, shows the importance of p16 as a prognostic and predictive biomarker and therapeutic target for cancer and metastasis.

Aside from p16 in cancer, although only p21-positive cells were able to prevent radiation-induced osteoporosis [86], p16 deletion inhibited oxidative stress, osteocyte senescence, and osteoclastic bone resorption, which led to osteogenesis and osteoblastic bone formation, indicating a promising mechanism to prevent estrogen deficiency-induced osteoporosis [137]. Furthermore, p16 deletion promoted migration, proliferation, and differentiation of bone marrow mesenchymal stem cells (BM-MSCs) and chondrocytes. It also stimulated osteoblastogenesis and vascularization, which improved bone fracture healing. Consequently, p16 modification might offer a novel strategy for treating fractured bones in elderly patients [138] (Figure 3 and Table 1).

### 4.3. In the Lungs

Cellular senescence and aging have both been linked to increased lung damage and functional impairment [139]. Growing evidence suggested aging as another determinant of the chronic obstructive pulmonary disease (COPD) and showed higher prevalence of the disease in elderly [140,141,142]. Similarly, even though there are no certain causes of idiopathic pulmonary fibrosis (IPF), aging associated with cellular senescence and p16 overexpression has emerged as a main risk factor [143,144].

Cigarette smoking (CS) is a major risk factor attributed to COPD [145]. CS can alter cellular proliferation and induce apoptosis, reactive oxygen species production, and promote oxidative stress, cause DNA damage, and trigger cellular senescence [146,147]. Furthermore, mice exposed to chronic cigarette smoking at both young and old ages showed increased activation of the senescence marker beta-galactosidase as well as upregulation of p16 compared to their respective air-exposed controls. Older air-exposed mice had higher levels of beta-galactosidase and p16 than younger mice. Therefore, CS-induced senescence and natural-aging-associated senescence are both affected by the p16 pathway [148]. This was confirmed in human COPD patients who had higher p16 expression compared to normal smokers and nonsmokers [149]. Furthermore, after CS exposure, wild type mice had more senescent alveolar type II (AECII) epithelial cells than p16 knockout mice, which had normal pulmonary function. Moreover, p16 deletion has rescued the adverse effects induced by CS in the lungs via the insulin growth factor1 (IGF1)/Akt1 signaling pathway [149].

However, p16 expression is a differentiation key between cervical squamous cell carcinoma (SCC) with pulmonary metastasis and pulmonary SCC. Immunohistochemistry of both cervical SCC without and with pulmonary metastasis has shown an intense staining of p16 in almost all cases studied. On the contrary, cases with pulmonary SCC demonstrated p16 expression in 7 out of 33 cases, 3 of which showed weak p16 staining. This implies the usefulness of p16 as a distinguishing marker between cervical SCC with lung metastasis and pulmonary SCC [150]. Furthermore, the fact that aberrant p16 methylation occurs at early stages of lung cancer renders p16 an early diagnostic biomarker for monitoring and prevention [151]. Moreover, p16 low expression and gene mutation were associated with early and late stage nonsmall cells lung carcinoma (NSCLC), respectively [152,153]. As a result, it has been identified as a predictable prognostic factor in NSCLC, particularly at the early stage.

On the other hand, p16 expression is not only linked with disease progression but also with lung protection. P16 loss was linked with poor survival after lung injury. In addition, p16 expression was found to be crucial for protection of lung epithelium against oncogenic stress and lung injury [154]. Moreover, injured p16-positive mesenchymal cells enhanced epithelial progenitor proliferation, whereas deletion of p16 attenuated normal epithelial repair in the lungs [155]. Furthermore, prevalent usefulness was demonstrated for p16 as a target for COPD therapy. Higher p16 expression was found in human COPD lungs compared to normal patients, and when CS induced impaired pulmonary function and augmented emphysema in WT mice, p16 knockout mice exhibited normal pulmonary function with reduced emphysema and increased alveolar progenitor proliferation [149] (Figure 3 and Table 1).

### 4.4. In the Brain

Aging-induced p16 overexpression and cellular senescence have been linked to decreased subventricular zone progenitor proliferation and neurogenesis of the olfactory bulb and to diminished multipotent progenitor cell frequency and self-renewal potency [156]. Moreover, chronic accumulation of senescent cells and the resulting inflammation in the brain has been linked to the development of Alzheimer’s disease (AD) and other neurodegenerative diseases [157,158]. In two out of five AD models, Dorigatti et al. [159] found evidence of cellular senescence marked by a significant increase in p16, p21, and p53 expression, as well as increased SASPs expression and beta-galactosidase activity [159]. Another study found that tau-containing neurofibrillary tangles (NFTs), a hallmark of Alzheimer’s disease, are age-dependent and strongly associated with senescence induction and upregulation of p16 and p21 [160,161]. Nonetheless, astrocytes play an important role in neuronal homeostasis and functions, and, as we age, they undergo senescence in response to multiple stresses, resulting in impaired brain function [162,163,164,165]. In a study to investigate the presence of senescent astrocytes in aging and Alzheimer’s disease, tissue from the brains of elderly people and Alzheimer’s patients was compared for p16 and SASPs expression with fetal tissue as a control. The findings revealed that aged brain tissue contains significantly more p16-positive astrocytes than fetal tissue. When compared to non-AD adults of the same age, AD brain tissue contains more p16-positive astrocytes [166].

As previously discussed for other tumors, unsurprisingly, p16 homozygous deletion was found in both primary glioblastoma and their derived xenografts [167]. In addition, p16-cdk4/cyclin D1-pRb pathway inactivation was found in the majority of glioblastomas [168]. P16 loss was linked to significantly poor outcome in all glioma patients, which indicates a predictive prognostic usefulness of p16 in brain tumors [169]. On the contrary, p16 null glioma cells demonstrated higher chemosensitivity to paclitaxel and topotecan compared to exogenous wild type p16 overexpression [170].

P16 overexpression has been shown to exert a protective function of neurons against CDK overexpression-induced apoptosis [171]. Moreover, increased expression of p16 and p21, induced by stress conditions, has protected female but not male astrocytes from transformation [172]. In another promising strategy, the selective elimination of p16-positive senescent astrocytes diminished cognitive impairment induced by whole brain irradiation [173]. Lastly, dihydromyricetin (DMY), through the downregulation of p16, p21, and p53, was able to inhibit oxidative stress and neuroinflammation and to attenuate brain aging and improve cognitive function in mice [174] (Figure 3 and Table 1).

### 4.5. In the Heart

Remarkable p16 expression and cellular senescence were found in cardiac chronological aging and heart failure [175,176]. For example, elevated p16 expression and beta-galactosidase activity were found in cardiomyocytes gathered from Langendorff heart perfusion with aging [91]. In addition to that, cardiac progenitor cells isolated from elderly (> 70 years old) people expressed high levels of p16 and SASPs, alongside shortened telomeres and increased SA-β-gal [92]. Furthermore, remarkable telomere shortening and senescent-associated increased p16 expression were found in cardiomyocytes isolated from old rats compared to younger ones [177]. Older patients with heart failure had higher p16 expression, which was associated with senescence and cell death, as well as shorter telomere length, when compared to healthy elderly people. This suggests that p16-induced senescence, telomere attrition, and cell death are features of heart failure in aging [176]. Furthermore, vascular smooth muscle cell (VSMC) senescence in atherosclerotic plaques was marked by increased p16, p21, and p53 expression in addition to increased beta-galactosidase activity [178].

The recovery of cardiac function and cardiac remodeling have been correlated with cardiac stem cell (CSCs) regeneration and differentiation ability [179,180]. Cellular senescence has an impact on CSCs and cardiac function, which might provide a concept of therapies by targeting senescent cells for cardiac functional improvement and extended lifespan in elderly people [179]. With aging, a significant portion of human CSCs become senescent with elevated expression of p16, SA-β-gal, and SASPs, which contribute to CSC senescence and impaired cardiac regeneration. However, INK-ATTAC or senolytic elimination of senescent CSCs reactivated resident CSCs and increased cardiomyocyte proliferation [179] reflecting the importance of p16-positive senescent CSCs as therapeutic approach for cardiac functional improvement. P16-positive cells that accumulate during adulthood have a negative impact on lifespan and promote age-dependent changes in the heart. The removal of p16-positive cells delayed age-related heart deterioration. Thus, the therapeutic removal of these cells may be an appealing approach to extend healthy lifespan [8].

On the contrary to the previous studies, the existence of p16 high cells detected in p16-CreERT2-tdTomato mouse model, was found indispensable for health span, and their elimination has induced cardiac fibrosis [83]. Furthermore, p16 overexpression has been detected in the infarction zone after myocardial infarction. The increased expression of p16 was associated with protected cardiac function and plays an important role for cardiac remodeling after myocardial infarction [181] (Figure 3 and Table 1).

### 4.6. In the Kidney

Several studies have linked p16 induction and subsequent cellular senescence to renal aging, diseases, and allograft rejection [182,183,184]. Age-dependent p16 upregulation in cortical tubular and interstitial cells was observed in humans. In addition, p16 and p27 expression were higher in the glomeruli, tubules, and interstitial cells of rejected grafts compared to normal kidneys [183]. Whether this reflects senescence as the underlying mechanism for chronic allograft rejection as suggested or might correspond to reduced proliferation and repair or to an increased immune reaction remains to be determined. In line with this, in human kidney specimens ranging from 8 weeks to 88 years of age, p16 induction was negatively correlated with the proliferation marker Ki-67 [185], which is in agreement with the role of p16 as a cell cycle inhibitor. Levels of p16 in glomerular and interstitial cells were significantly higher in kidneys with glomerular disease than in normal aged kidneys and kidneys with tubular interstitial nephritis. P16 expression was higher in kidneys with proteinuria, with fibrosis, or interstitial inflammation [186]. Whether this increased P16 expression is cause or consequence of glomerular disease remains an open question. Similarly, increased p16 expression was observed in kidneys of hypertensive animals and patients and kidneys with type 2 diabetic nephropathy [187,188]. Blood pressure lowering reduced p16 expression [187], which argues against a close relation between P16 and irreversible senescence in this model. Increased p16 expression has been reported in acute kidney injury (AKI) and in acute tubular necrosis (ATN) [189]. P16 deletion ameliorated ATN and improved kidney function in animal models [189]. Similarly, p16 deletion in Bmi-1-deficient mice rescued kidney aging features including function and structure, ameliorated tubulointerstitial fibrosis, and inhibited epithelial mesenchymal transition of renal interstitial fibroblasts [190] (Figure 3 and Table 1).

### 4.7. In the Liver

Although the majority of liver functions appear to be preserved with age, evidence of aging and cellular senescence associated with liver functional decline, reduced regenerative capacity, and diseases are well-documented [191,192,193]. P16 expression was higher in elderly hepatectomy patients compared to younger ones, and the increased p16 expression was associated with decreased liver regeneration [194]. This is in agreement with the attenuated proliferative response of hepatocytes in old rat liver compared to young animals [195]. P16 upregulation was observed in liver tissue and liver sinusoidal endothelial cells (LSEC) in an aged rat model compared to young animals [196]. The p16 CreERT2 tdTomato mouse model also demonstrated that p16 high cells were detectable in the liver, and that they were enriched with aging. The majority of the P16-positive liver cells found were vascular endothelial, and their removal caused steatohepatitis and perivascular tissue fibrosis [83,197]. This is compatible with higher p16 expression level of liver endothelial cells compared to nonendothelial cells demonstrated in our recent study [120].

With respect to liver metabolism, the extra copy of p16 carried by the “Super-INK4A/ARF” mouse model prevented the development of glucose intolerance with aging. Instead, increased activation of insulin receptors and high insulin sensitivity were obtained. This reveals a protective role of INK4A/ARF locus against age-induced insulin resistance [198], whereas increased insulin secretion, attenuated insulin sensitivity, and reduced hepatic insulin clearance were observed upon loss of function mutation of the Cdkn2a gene [199]. On the contrary, p16 deficiency improved fasting-activated glucose production in the liver, via the activation of PKA-CREB-PGC1α [200]. Altogether, these studies show the importance of p16 in glucose homeostasis. However, p16 has not been only implicated in glucose but also in fat metabolism. P16 has been found to regulate fasting-induced fatty acid oxidation and lipid droplet accumulation in the liver in vivo and in vitro. In addition, p16 deficiency was correlated with increased expression of fatty acids catabolism genes in primary hepatocytes [201]. Furthermore, p16-positive senescent cell accumulation has been correlated with hepatic fat deposition and steatosis. Elimination of these cells in the INK-ATTAC mouse model or senolytics treatment (dasatinib plus quercetin) attenuated liver fibrosis [88]. However, the feedback loop between lipid accumulation and increased p16 expression remains intriguing. Senescence in hepatocytes triggered fat accumulation [88], while high fat diet provoked significantly elevated p16 expression [202]. A possible mechanism could be that due to high fat diet and with aging, increased p16 induces senescent cell accumulation in the liver which in turn impairs lipid metabolism and provokes liver fibrosis. Therefore, p16-positive senescent cells might be a promising target for liver fibrosis therapy. On the other hand, elimination of p16-positive cells also provokes liver fibrosis [83]. In primary sclerosing cholangitis, p16 downregulation demonstrated a protective effect against biliary damage and fibrosis [203]. However, its upregulation was required for the regulation of reactive oxygen species in hepatic stellate cells and modulation of liver fibrosis [204].

Nonetheless, several studies have also described p16 functions in liver cancers. P16 hypermethylation and consequent p16 inactivation has a pivotal role in the development of hepatocellular carcinoma and liver cirrhosis [205]. Wong et al. reported aberrantly methylated p16 in the plasma of liver cancer patients, suggesting the usefulness of these circulating liver-cancer-methylated DNA for the monitoring of tumors [206]. Therefore, all this information combined suggests that p16 regulation and meticulously unravelling the molecular mechanisms regulating p16 expression in liver physiology and liver pathologies require further elucidation and could unveil novel therapeutic strategies for maintaining normal liver function and extending lifespan (Figure 3 and Table 1).

**Table 1:** Major p16 functions in the variety of biological processes observed in different models and tissues.

Process	Model and/or Tissue	Potential Role/Function	References
**Intervertebral disc damage**	Mouse and human intervertebral disc tissues	P16 is a potential therapeutic target for intervertebral disc damage relief.	[48]
**Wound healing**	P16-3MR model (fibroblasts, endothelial cells, and keratinocytes)	Accelerate wound closure and re-epithelialization	[43]
**Tumorigenesis and tumor suppression**	Skin, bone, lung, liver, and brain cancer patients tissues and immunohistopathology	Implicated in tumor development, progression, and metastasis Predictive and prognostic marker Therapeutic target Increases chemosensitivity	[39,40,41,42,72,105,106,107,108,111,112,134,135,136,150,151,152,153,168,169,170,205,206]
**Stem cell self-renewal and differentiation**	Skin, lung, bone, brain, and heart stem cells	Balanced expression of p16 is a prerequisite for stem cells proliferation and differentiation. Therapeutic approach for maintenance of regenerative capacity	[46,92,101,115,116,117,118,119,138,149,155,156,179]
**Cellular senescence**	Primary mouse fibroblasts and melanocytes	Target for oncogene-induced senescence bypass and aging	[37,38]
**Bone homeostasis**	P16-3MR and p16-INK-ATTAC mouse model	Maintenance of bone mass Orchestration of osteoblast and osteoclast function	[123,132,133]
**Bone fracture healing**	Geriatric Mouse model (p16-/- and WT)	P16-deletion stimulated osteoblastogenesis and vascularization and accelerated bone fracture healing	[138]
**Muscle injury**	Acute muscle injury (AIM) mouse model	Tissue regeneration	[45]
**Osteoporosis**	Ovariectomized p16-/- and WT mice	Potential therapeutic target to prevent estrogen-induced osteoporosis	[124,125,126,127,137]
**COPD**	Lung alveolar and lung epithelial cells in mice and human	Implicated in COPD severity Potential therapeutic target	[140,141,142,149]
**Cervical SCC and pulmonary SCC**	Human cancer patients	Discriminating biomarker	[150]
**Oxidative stress**	Fibroblasts, keratinocytes, and melanocytes	P16 regulates oxidative stress and ROS production as pRB-independent tumor suppression mechanism	[72,204]
**Mitochondrial biogenesis**	Primary mouse fibroblasts, human melanocytes, A375 melanoma cells	P16 balances mitochondrial structure and function	[111]
**Alzheimer’s disease (AD)**	Alzheimer’s disease patients and mouse model	Implicated in AD severity and development Therapeutic target	[159,160,161,166]
**Lung injury**	P16-/- and WT mouse model (lung epithelium)	P16 protects against lungs injury	[154]
**Cardiac fibrosis**	p16-CreERT2-tdTomato mouse model	P16-positive cells removal induces cardiac fibrosis	[83]
**Myocardial Infarction**	Mice	Indispensable for maintenance of cardiac function and cardiac remodeling after infarction	[181]
**Glucose metabolism and homeostasis**	Super-INK4A/ARF mice model	Prevented the development of glucose intolerance with aging Protective role against age-induced insulin resistance	[198]
**Liver fibrosis**	INK-ATTAC mouse model	Therapeutic approach for treatment of liver fibrosis	[83,88,204]
**Fat metabolism**	Mouse model and primary hepatocytes	Regulate fasting-induced fatty acid oxidation and lipid droplet accumulation in the liver	[201]
**Development**	Young mice brain, heart, kidney, and liver	Dynamic p16 expression detected in embryonic mice organs, reflecting a potential role in embryonic development	[120]

## 5. The Role of p16 in Development

The role of p16 and cellular senescence in embryonic development is still controversial, which was reviewed recently [207]. Earlier studies reported p21 expression during embryonic development, but p16 and p19ARF were not detected [54,208]. Cellular senescence has been described as a normal developmental mechanism, and senescent cells were observed in distinct locations and time windows in the developing embryo through beta-galactosidase staining. However, only p21 expression together with other senescence markers were detected, but p16 and p19ARF were undetectable [51,52]. Additionally, in an attempt to study cellular senescence in rat kidney with aging, negative p16 expression was reported in young rats [209]. On the other hand, in embryonic mice, p19ARF-induced senescence has been associated with neurodevelopmental abnormalities [210]. Furthermore, p19ARF expression has been implicated in nervous system development [53]. Interestingly, mice deficient for p16 and p19ARF exhibited cataract formation as developmental defects in the eye [211]. Moreover, p16 expression was observed in embryonic and postnatal rat brain and embryonic mouse motor neurons [212,213].

Although several studies could not detect p16 expression in embryonic life, the authors, however, did not exclude expression of p16, but the absence of detection might have been due to technical limitations [53,54]. Therefore, we used highly sensitive RT-qPCR and immunohistochemistry methods [214,215,216,217,218] in our recent study conducted in mice at embryonic (E10-E18), postnatal (P1-P21), adult (3 months), and old (16–18 months) ages to investigate p16 expression in the brain, heart, kidney, and liver. P16 RNA and protein expression level were assessed, beside p19ARF and p21 RNA expression. Our results demonstrated a dramatic increase of p16 expression at RNA and protein level in old mice compared to younger ages, which is in agreement with other studies describing p16 as an aging marker [5,7,30,219,220,221,222]. Less pronounced upregulation of p19ARF and p21 in old mice compared to p16 was obtained. Nevertheless, the assessment of Il-6, Mmp-9, Tgf-β1, and Vegfa expression as SASP factors has also shown an increased expression of the four genes with aging in all investigated organs [120]. Interestingly, at embryonic and postnatal ages, our results revealed a relatively high and dynamic expression of p16 with a constant increase in all organs in a matter of days, whereas p19ARF and p21 expression levels showed less remarkable variations (Table 1). In addition, SASPs mRNA expression showed a baseline level and did not correlate with p16 at embryonic stages [120] implying that p16 expression during development does not necessarily refer to cellular senescence [223]. Afterwards, our immunohistochemistry results indicated strong vascular signal of p16 expression in adult and old tissue. Therefore, we intended to conduct a comparative p16 expression assessment between endothelial and nonendothelial in all organs studied from adult and old mice. Surprisingly, q RT-PCR and Western blot revealed significantly higher p16 in old liver endothelial cells compared to liver parenchymal cells, which is also in agreement with what was detected in the p16-Cre^ERT^-tdTomato mouse model [83].

These results suggest dynamic p16 expression and a potential role in embryonic development. A p16-knockout model or the p16 ablator mouse strains for phenotype observations might be a promising strategy for unveiling the role of p16 in development. In addition, p16 is specifically upregulated in endothelial cells in the liver of aged animals, suggesting a selective role of p16 in endothelial cells of the liver. Therefore, further elucidation of the p16 mechanism in development and liver physiology is required.

## 6. Conclusions

Most of the different roles and pathological conditions described above are in general agreement with the basic function of p16 as a negative regulator of the cell cycle. Elevated P16 expression in cells during development coincides with the beginning of differentiation. For this, lower proliferation is a prerequisite. Additionally, tumor suppression is a logical consequence of cell cycle inhibition. Senescence in old age with dramatically increased p16 expression could be viewed as an extreme case of cell cycle inhibition. In many preclinical models, removal of senescent cells or modification of the SASP showed beneficial effects, and first clinical trials with senolytics are promising (reviewed in [224,225]). Nevertheless, different p16 ablator mouse models also showed opposite effects on health span. Thus, it would be highly interesting to directly compare the SASPs of these different models, which might open the way to target the divergent SASP factors for improved health outcome in old people. Finally, the high p16 expression, especially in endothelial cells in old age, and the fact that elimination of these p16^high^ cells causes liver damage needs further functional elucidation.
